# Clinical Improvement of Club Foot with Osteopathic Manipulative Treatment in the Peruvian Amazon

**DOI:** 10.51894/001c.165996

**Published:** 2026-07-31

**Authors:** Cydney Pittenger, Vidhula Srinivasan, Travis Gordon

**Affiliations:** 1 College of Osteopathic Medicine Michigan State University

## 113

### Introduction

Clubfoot (talipes equinovarus) is a congenital deformity affecting 1 in 1,000 newborns, characterized by hindfoot equinus and varus, midfoot cavus, and forefoot adduction. Standard management includes serial casting and bracing, physical therapy, and, in refractory cases, surgical intervention. Some patients experience persistent functional limitation, possibly developing psychological distress due their impaired mobility. Literature remains limited, but osteopathic manipulative treatment (OMT) may serve as an adjunct.

### Case Description

A 17 year-old male presenting to an annual MSUCOM outreach initiative in Iquitos, Peru revealed a history of congenital left club foot, status-post Achilles tenotomy and left anterior tibialis tendon transfer at 11 months. The patient reported chronic pain and limited range of motion, restricting his ability to run, play soccer, and engage with peers, leading to social exclusion and psychological distress.

Physical exam revealed valgum deformity of the left foot with significantly restricted ROM in plantar flexion. Significant left leg atrophy was present with 8cm circumferential difference noted. The left leg was 2cm shorter than the right leg. Osteopathic structural exam revealed somatic dysfunctions of the lower extremity, pelvis, visceral and cranial regions. Balanced ligamentous tension (BLT), osteopathic cranial manipulative medicine (OCMM), and high velocity low amplitude (HVLA) were used to treat dysfunctions.

The left lower extremity interosseous membrane was determined to be the key dysfunction. Its treatment with BLT resulted in a brief still point and subsequent prolonged primary respiratory cycle lasting 10 minutes and 40 seconds, followed by correction of a left temporal bone dysfunction. High-velocity, low-amplitude (HVLA) technique corrected anterior talus dysfunction and left leg length discrepancy. The patient reported immediate pain reduction, ability to ambulate without discomfort, and significant improvement in left foot and ankle range of motion.

### Discussion

This case highlights the potential role of OMT in addressing chronic pain, biomechanical dysfunction, and associated psychological distress related to congenital clubfoot. Significant immediate changes in ROM, biomechanics and pain were noted, however, further follow-up was not possible given the outreach nature of our visit to Peru. Further research of OMT to treat clubfoot is warranted to explore its role as an adjunctive treatment.

